# Feasibility of Onchocerciasis Elimination Using a “Test-and-not-treat” Strategy in *Loa loa* Co-endemic Areas

**DOI:** 10.1093/cid/ciaa1829

**Published:** 2020-12-08

**Authors:** David J Blok, Joseph Kamgno, Sebastien D Pion, Hugues C Nana-Djeunga, Yannick Niamsi-Emalio, Cedric B Chesnais, Charles D Mackenzie, Amy D Klion, Daniel A Fletcher, Thomas B Nutman, Sake J de Vlas, Michel Boussinesq, Wilma A Stolk

**Affiliations:** 1 Department of Public Health, Erasmus MC, University Medical Center, Rotterdam, The Netherlands; 2 Centre for Research on Filariasis and other Tropical Diseases (CRFilMT), Yaoundé, Cameroon; 3 IRD UMI 233-INSERM U1175-Montpellier University, Montpellier, France; 4 Task force for Global Health, Decatur, Georgia, United States; 5 Laboratory of Parasitic Diseases, National Institute of Allergy and Infectious Diseases, Bethesda, Maryland, United States; 6 Department of Bioengineering and the Biophysics Program, University of California, Berkeley, California, United States

**Keywords:** onchocerciasis, *Loa loa*, point-of-care testing, elimination, modeling

## Abstract

**Background:**

Mass drug administration (MDA) with ivermectin is the main strategy for onchocerciasis elimination. Ivermectin is generally safe, but is associated with serious adverse events in individuals with high *Loa loa* microfilarial densities (MFD). Therefore, ivermectin MDA is not recommended in areas where onchocerciasis is hypo-endemic and *L loa* is co-endemic. To eliminate onchocerciasis in those areas, a test-and-not-treat (TaNT) strategy has been proposed. We investigated whether onchocerciasis elimination can be achieved using TaNT and the required duration.

**Methods:**

We used the individual-based model ONCHOSIM to predict the impact of TaNT on onchocerciasis microfilarial (mf) prevalence. We simulated precontrol mf prevalence levels from 2% to 40%. The impact of TaNT was simulated under varying levels of participation, systematic nonparticipation, and exclusion from ivermectin resulting from high *L loa* MFD. For each scenario, we assessed the time to elimination, defined as bringing onchocerciasis mf prevalence below 1.4%.

**Results:**

In areas with 30% to 40% precontrol mf prevalence, the model predicted that it would take between 14 and 16 years to bring the mf prevalence below 1.4% using conventional MDA, assuming 65% participation. TaNT would increase the time to elimination by up to 1.5 years, depending on the level of systematic nonparticipation and the exclusion rate. At lower exclusion rates (≤2.5%), the delay would be less than 6 months.

**Conclusions:**

Our model predicts that onchocerciasis can be eliminated using TaNT in *L loa* co-endemic areas. The required treatment duration using TaNT would be only slightly longer than in areas with conventional MDA, provided that participation is good.

## Teaser

Our model predicts that onchocerciasis can be eliminated using test-and-not-treat (TaNT) in *L loa* co-endemic areas. The required treatment duration using TaNT would be only slightly longer than in areas with conventional mass drug administration, provided that participation is good.

Mass drug administration (MDA) with ivermectin is the main strategy for eliminating onchocerciasis. Although ivermectin is generally safe, the drug has been associated with serious adverse events (SAE) in persons with *Loa loa* [1], a filarial parasite endemic in forest areas of Central Africa [2]. People with *L loa* microfilarial densities (MFD) greater than 30 000 microfilariae per milliliter (mf/mL) of blood are at high risk of developing potentially fatal encephalopathy [3, 4]. Since the 1990s, more than 500 SAE cases with encephalopathy have been reported after treatment with ivermectin, of which 60 led to death [4, 5].

The World Health Organization/Mectizan Donation Program guidelines approve ivermectin MDA in meso- and hyperendemic onchocerciasis areas (ie, onchocercal nodule prevalence >20% in adults) with loiasis co-endemicity, if accompanied by enhanced surveillance for adverse events [6]. The potential benefits of MDA (eg, prevention of blindness and onchodermatitis) were felt to outweigh the potential risk of *Loa*-related post-ivermectin SAEs, and early supportive care of SAE cases could reduce the risk of mortality and long-term sequelae. However, there is no World Health Organization-endorsed strategy for hypoendemic onchocerciasis areas with loiasis co-endemicity, which hinders onchocerciasis elimination.

To eliminate onchocerciasis in those areas, a test-and-not-treat (TaNT) strategy has been proposed [5]. In a pilot study in Cameroon, the LoaScope (a mobile video microscope [7]) was used to rapidly identify individuals with a loiasis MFD of ≥ 20 000 mf/mL, who were then excluded from subsequent ivermectin treatment [5]. In this study, the risk threshold was lowered for safety reasons. The participation rate in health areas varied between 51.5% and 68.4% of the total population, which was considered acceptable given the history of post-ivermectin loiasis-related SAEs in 1999 (after which ivermectin distribution was interrupted in hypoendemic onchocerciasis areas). Overall, only 2.1% of the subjects tested with the LoaScope were excluded from ivermectin treatment because of high loiasis MFD. During the second TaNT campaign in 2017, this percentage dropped to 1.5%. Of the individuals treated with ivermectin in the first round, 99.97% remained below the risk threshold, indicating that those individuals could have been safely retreated without retesting [8].

Although the TaNT strategy has been successfully piloted in Cameroon, it remains unclear whether elimination of onchocerciasis is possible using this strategy and how long the program would have to be continued. We address these questions by mathematical modeling. For this purpose, we used the individual-based model ONCHOSIM [9, 10], which has been used previously to predict the impact of mass treatment on infection trends and to estimate the required treatment duration for achieving elimination [10–12].

## METHODS

### Model

ONCHOSIM is a stochastic individual-based model that simulates the transmission of onchocerciasis in a closed dynamic population of approximately 440 individuals (rural village) [13, 14]. The model simulates life histories of human individuals and *Onchocerca volvulus* worms and mf within individual human hosts. Transmission of infection occurs through bites of blackflies, whose intensity is represented by the annual biting rate. The probability that an individual is bitten by a blackfly is assumed to depend on age (exposure to blackfly increases linearly between the ages of 0 and 20), sex (higher exposure in males), personal factors (eg, attractiveness to blackflies), and seasonal biting variation of blackflies. At each bite, blackflies can transmit or pick up the infection. Only a small proportion of transmitted larvae will successfully develop into adult worms. Following insemination of females by male worms, mf are produced, which can be picked up by the blackfly. These mf develop in the blackfly into the infective stage (L3), which is modeled deterministically in the vector. Infection acquired from other villages is captured by the parameter called external force of infection. Our model did not account for an association between onchocerciasis and loiasis intensity within individuals because evidence suggests that the association is weak and insufficient to explain very high loiasis MFD [15]. Supplementary Table S1 provides information about the quantification of biological parameters.

### Precontrol Setting

We simulated hypoendemic precontrol *O volvulus* mf prevalence between 2% and 40% by varying setting-specific transmission parameters: the annual biting rate, the shape of the gamma distribution describing variation in exposure between individuals, and the level of external force of infection. Parameter values were sampled from a predefined parameter space and were accepted when the resulting precontrol mf prevalence in the endemic equilibrium would fall into 1 of the bins: 2% and 5%, 5% and 10%, 10% and 15%, 15% and 20%, 20% and 25%, 25% and 30%, 30% and 35%, and 35% and 40%. The model was run until we had 1000 parameter combinations for each bin. The sampled parameter space and the underlying distribution of the intensity of infection are provided in Supplementary Figures S1 and S2.

### Treatment Scenarios

#### Test-and-not-treat Strategy

The modeled TaNT strategy includes annual testing with the LoaScope of individuals for high loiasis MFD, which is repeated during the entire period of the simulation. Individuals who tested negative (ie, loiasis MFD <20 000 mf/mL) were provided ivermectin. In our model, treatment with ivermectin was assumed to kill 99% of *O volvulus* mf within 1 month [16].

The probability of participation of individuals in TaNT rounds is determined by age, sex, and a lifelong participation factor. Children younger than age 5 years and a proportion of women in the reproductive age (pregnant or lactating) are excluded from TaNT, because they are not eligible to receive ivermectin. The lifelong participation factor (score between 0 and 1) represents personal circumstances that makes an individual less (low score) or more likely (higher score) to participate [9, 10]. Some individuals may never participate, because they are chronically ill or refuse treatment, and are represented by the proportion of systematic nonparticipation.

The participation rate is defined as the percentage of the total population that is tested per round. With lower participation rates, systematic nonparticipation would be more likely. To cover realistic scenarios, we defined 7 participation scenarios varying both the participation rate (50%, 65%, and 80%) and the systematic nonparticipation (0%, 5%, and 10%) (Supplementary Table S2).

As a result of TaNT, an additional percentage of the individuals tested is excluded from ivermectin because of high loiasis MFD. On average, 2.1% of those tested were excluded from ivermectin during the pilot TaNT campaign [5]. To capture possible heterogeneity across endemic areas, we varied the exclusion rate: 1%, 2.5%, 5%, 7.5%, and 10%. Because loiasis MFD in individuals has been found to be stable over time, we assume that individuals with high loiasis MFD remain excluded from ivermectin during their whole lifetime [17]. TaNT exclusion is modeled similarly to systematic nonparticipation.

In the main analysis, we assumed that the annual TaNT exclusion rate remains fixed. However, observations from the second TaNT round in Okola showed that the exclusion dropped from 2.1% to 1.5% within 18 months [8]. In a sensitivity analysis, we explored the impact of a decreasing exclusion rate. To match the observed change, we assumed an exponential drop (rate: 0.22 per year) in the first 10 TaNT rounds and no exclusion because of high loiasis MFD in the following rounds.

#### Mass Drug Administration

As a reference scenario, we explored the impact of conventional ivermectin MDA (ie, without pretesting), in which no one was excluded from treatment because of loiasis. Ivermectin MDA is simulated by specifying the time and coverage of the treatment. To compare this scenario head to head with the TaNT scenarios, we simulated annual ivermectin MDA using the same participation rates and systematic nonparticipation as in the TaNT scenarios.

### Analysis

For precontrol mf prevalence levels between 2% and 40%, we predicted the impact of TaNT in loiasis co-endemic areas and the impact of annual ivermectin MDA (reference scenario) over 25 years.

In our scenarios, we assume that treatment in the main village and neighboring villages are started simultaneously, resulting in a drop of the external force of infection over time. To estimate the decline in the external force of infection, we simulated a hyperendemic area with annual ivermectin MDA and used the modeled rate of decline in the force of infection as a proxy (Supplementary Figure S3).

For each treatment scenario, we assessed the time until elimination of onchocerciasis. As in previous studies, we defined elimination as reaching a modeled mf prevalence below 1.4% (ie, operational threshold for treatment interruption followed by surveillance) [9, 18]. We ran the model for 50 years and assessed the year in which the mf prevalence first fell below 1.4% per run. If the target was not met, we assumed a duration until elimination of 50 years (equals the simulated period). Per treatment scenario, we then calculated the mean required treatment duration per precontrol level. We only present the results over a period of 25 years.

For each treatment scenario, we also assessed the annual probability of achieving elimination, which was calculated as the proportion of runs in which the mf prevalence fell below 1.4% per year by precontrol setting.

Our study follows to the modeling principles of the NTD Modelling Consortium for policy-relevant work (Supplementary Table S3) [19].

## RESULTS

**Figure 1.**
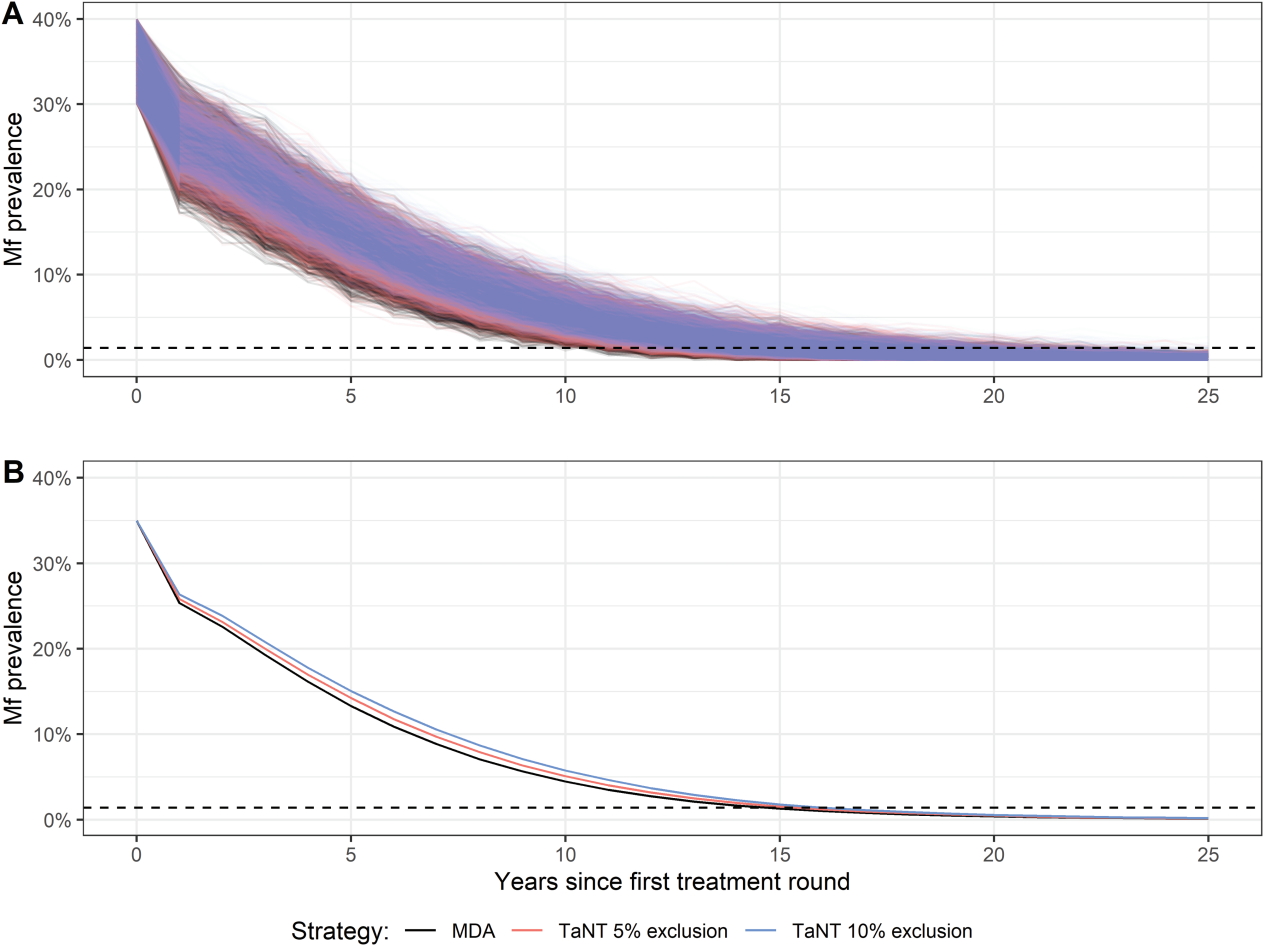
Trends of onchocerciasis mf prevalence in a setting with 30% to 40% precontrol mf prevalence assuming 65% participation with 5% systematic nonparticipation. The mf prevalence was calculated every year before the start of a TaNT round. Any changes within a year are not shown. *A*, Results of individuals runs, showing the variation between those runs. *B*, Mean mf prevalence for all runs. The black line represents the reference scenarios (ie, ivermectin MDA without pretesting). The red and blue lines represent the TaNT strategy assuming 5% and 10% exclusion because of high *Loa loa* MFD, respectively. We assume that TaNT is continued during the entire simulation period. The dashed line depicts the elimination threshold of 1.4% mf prevalence. Abbreviations: MDA, mass drug administration; mf, microfilarial; MFD, microfilarial densities; TaNT, test-and-not-treat.

Figure 1 shows the trend in onchocerciasis mf prevalence of the upper end of the hypoendemic profile (30%-40%), assuming 65% participation with 5% systematic nonparticipation. Using conventional ivermectin MDA, elimination of onchocerciasis could be achieved after an average of 15 years. Implementing the TaNT strategy, assuming 5% and 10% TaNT exclusion, prolongs the mean time until elimination by 0.7 and 1.4 years, respectively.

**Figure 2.**
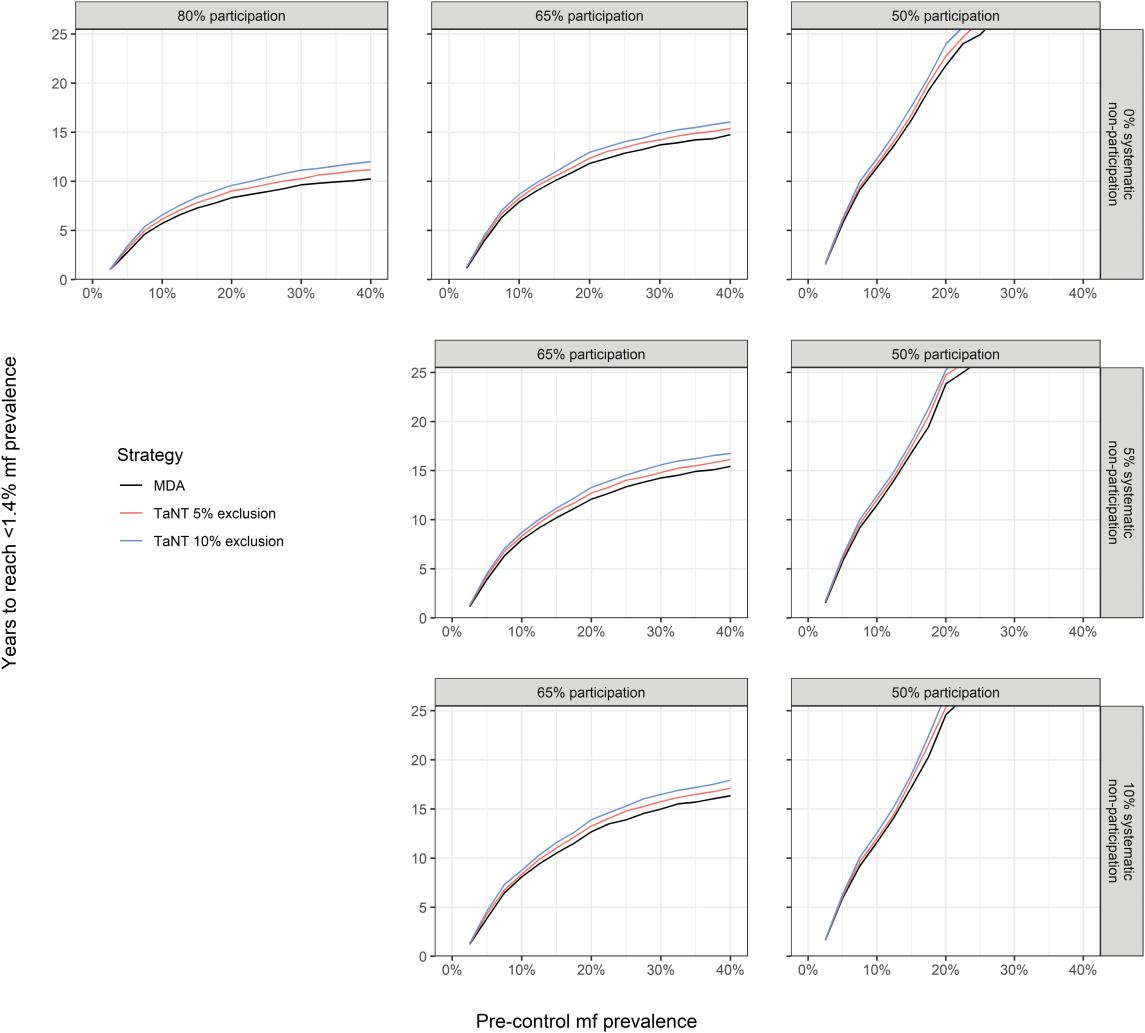
Average time needed to reach an onchocerciasis mf prevalence below 1.4% by precontrol level. The black line represents the reference scenarios (ie, ivermectin MDA) without pretesting. The red and blue lines represent TaNT assuming 5% and 10% exclusion from high *Loa loa* MFD, respectively. Each panel shows results under varying assumptions of participation (ie, 80%, 65%, and 50%) and systematic nonparticipation (ie, 0%, 5%, and 10%). Abbreviations: MDA, mass drug administration; mf, microfilarial; MFD, microfilarial densities; TaNT, test-and-not-treat.

The delay in achieving onchocerciasis elimination using a TaNT strategy compared with conventional ivermectin MDA depends on the precontrol onchocerciasis mf prevalence, participation rate, systematic nonparticipation, and TaNT exclusion (Figure 2). Areas with lower levels of precontrol mf prevalence, higher participation rates, and lower proportions of systematic nonparticipation would reach elimination sooner compared with areas with higher prevalence, lower participation, and higher systematic nonparticipation. Areas with a precontrol mf level above 25% would not reach elimination within 25 years if the participation rate were 50%. Elimination could be reached within 25 and 15 years if 65% and 80% of the population participates, respectively.

Table 1 summarizes the average time to elimination for all treatment scenarios. Higher precontrol mf levels increase the time to onchocerciasis elimination by 2- to 5-fold compared with 2% to 10% precontrol mf prevalence. A 65% and 50% participation would increase the time to elimination by 1.4-fold and >2-fold compared with 80% participation, respectively. The time to elimination increases with higher TaNT exclusion rates. This increase is larger for areas with high precontrol onchocerciasis mf prevalence levels and high levels of systematic nonparticipation. At an exclusion of 10%, the time to elimination increases by 1.7, 1.5, and 3 years at most, assuming an 80%, 65%, and 50% participation, respectively. At lower levels of TaNT exclusion (ie, ≤2.5%), the delay to reach the assumed elimination threshold compared with ivermectin MDA varies between 0.0 and 0.4, 0.0 and 0.3, and 0.1 and 0.8 years, assuming an 80%, 65%, and 50% participation, respectively.

**Table 1.**
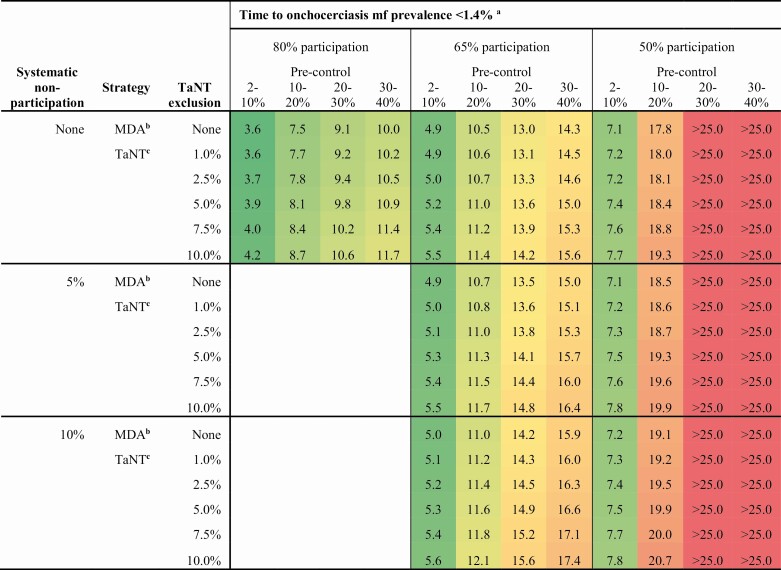
Average Time Needed to Reach anOnchocerciasis mf Prevalence Below 1.4% by Precontrol mf Prevalence Level

Abbreviations: MDA, mass drug administration; mf, microfilarial; TaNT, test-and-not-treat.

^a^Mean duration to reach the elimination threshold per precontrol bin; the colors indicate the duration: gradient from green (short duration) to red (long duration).

^b^Ivermectin mass drug administration without pretesting (ie, no exclusion).

^c^Test-and-not-treat strategy.

**Figure 3.**
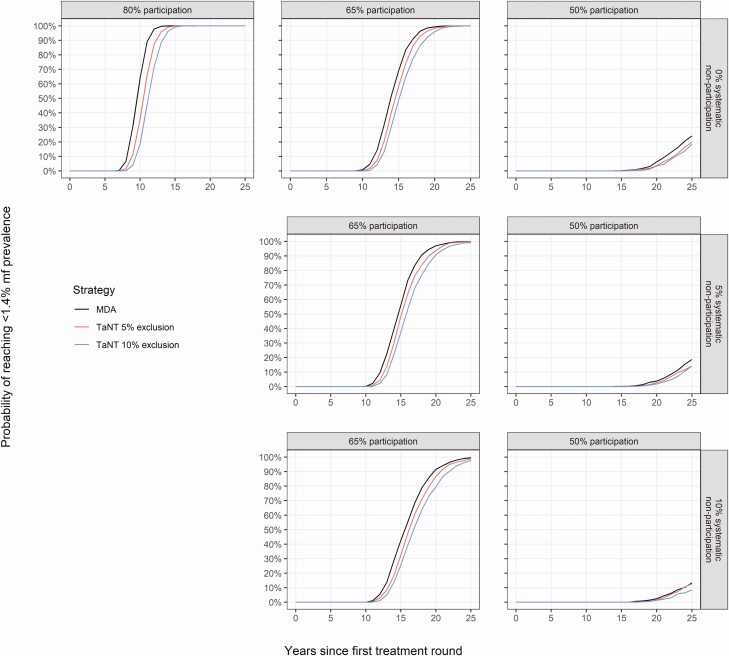
Probability of reaching an onchocerciasis mf prevalence below 1.4% in a setting with 30% to 40% precontrol mf prevalence. The black line represents the reference scenarios (ie, ivermectin MDA) without pretesting. The red and blue lines represent TaNT assuming 5% and 10% exclusion from high *Loa loa* MFD, respectively. Each panel shows results under varying assumptions of participation (ie, 80%, 65%, and 50%) and systematic nonparticipation (ie, 0%, 5%, and 10%). Abbreviations: MDA, mass drug administration; mf, microfilarial; MFD, microfilarial densities; TaNT, test-and-not-treat.

The probability of achieving onchocerciasis elimination increases with TaNT rounds in areas with 30% to 40% precontrol mf prevalence (Figure 3). Reaching elimination within 25 years is very likely if the participation is 80% or 65%, and very unlikely if the participation is 50%. Lower rates of systematic nonparticipation and TaNT exclusion would increase the probability of reaching the elimination threshold.

**Figure 4.**
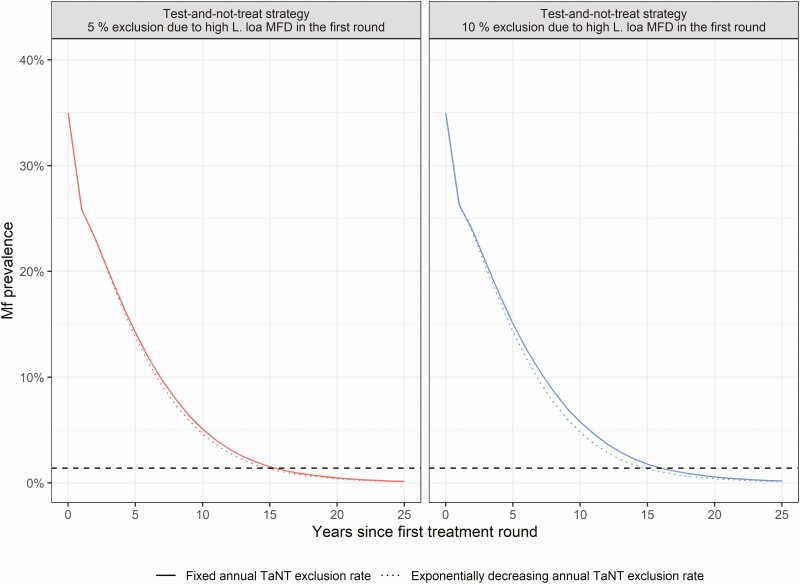
Trend of onchocerciasis mf prevalence in a setting with 30% to 40% precontrol mf prevalence assuming a fixed versus a decreasing annual exclusion percentage. The solid line represents the scenario assuming a fixed annual exclusion rate from high *Loa loa* MFD during all treatment rounds. The dotted line represents the scenario assuming an exponentially drop in the annual exclusion with a rate of 0.22 per year in the first 10 years, followed by no exclusion afterwards. The red and blue colored lines represent TaNT assuming an initial exclusion rate of 5% and 10% from high *L loa* MFD, respectively. The horizontal dashed line is the onchocerciasis elimination threshold. Abbreviations: MDA, mass drug administration; mf, microfilarial; MFD, microfilarial densities; TaNT, test-and-not-treat.

When we assume that the TaNT exclusion rate decreases over time, the time to elimination would be 0.6 and 1.3 years shorter (assuming an initial exclusion rate of 5% and 10%, respectively) compared with a fixed exclusion rate (Figure 4).

## DISCUSSION

This study suggests that onchocerciasis can be eliminated in *L loa* co-endemic areas using a TaNT strategy provided that participation is good. In areas with 30% to 40% precontrol mf prevalence, it would normally take approximately 14 to 16 years to bring onchocerciasis mf prevalence below 1.4% using conventional ivermectin MDA, assuming 65% participation. A TaNT strategy would increase the duration of reaching elimination by only 1.5 years if 10% of the population were excluded from ivermectin treatment. At lower exclusion rates (≤2.5%) the delay would be less than 6 months, which is very promising. The most challenging areas will be those that are in the upper end of the hypoendemic profile. In these areas, the delay is generally longer and therefore more TaNT rounds and a higher participation would be required.

Our results support the notion that good participation with minimal systematic nonparticipation is essential to eliminate onchocerciasis [11]. This holds true for both conventional ivermectin MDA and TaNT. A 65% participation rate of the total population may be considered acceptable but increasing it to 80% would further shorten the duration of elimination programs, especially in areas with precontrol onchocerciasis mf prevalences of ≥20%. If participation were as low as 50%, it is very unlikely that onchocerciasis would be eliminated within 25 years, especially if a large proportion of the population is systematically not treated (because of systematic nonparticipation or TaNT exclusion). Reaching high participation for TaNT might be more difficult than for ivermectin MDA because blood samples need to be taken during daytime hours because of the diurnal periodicity of *L loa* mf in the peripheral blood, making TaNT less flexible logistically [20]. In the pilot TaNT campaign, the participation varied between 51.5% and 68.4% of the total population [5]. Low participation was believed to be the result of fear of SAEs based on experiences with neurological SAEs and deaths. In the second round, participation was higher and varied between 60.5% and 78% of the total population [8]. Another TaNT campaign conducted in 2017-2018 in the neighboring Soa health district and involving local health personnel and community volunteers showed an overall participation of approximately 66% in rural settings [21]. Urban and semiurban areas show consistently lower rates of participation [5, 21]. The rationale of getting tested for safe treatment might lead to higher willingness to participate in future rounds, which could further reduce the time until elimination. Involvement of local personnel and volunteers might add trust and participation. Also, health education strategies have been suggested to increase participation [22].

To reach the assumed elimination threshold, our main findings suggest that TaNT should be repeated for 14.5 to 17.4 years in areas with precontrol onchocerciasis mf levels 30% to 40% and 65% participation. In Okola (precontrol mf prevalence: 15.3%-29.9%) [5], the time to elimination would range between 11.3 and 14.6 years assuming 65% participation and 2.5% exclusion. Although sustaining TaNT for a long period would create a burden on healthcare systems in terms of operational challenges and costs, it is encouraging that the present study predicts that TaNT would delay elimination by a maximum of only 1.5 years. Moreover, it is very likely that the proportion that needs to be excluded because of high loiasis MFD will decrease over time. If an optimistic decreasing rate of exclusion is assumed, this could reduce the time to elimination by up to 1.3 years (Figure 4). To reduce the operational challenges and cost of TaNT, testing only those who were not tested previously or were excluded because of high loiasis MFD should be considered. After several years, the number of individuals requiring testing would be small. Conventional MDA could be organized for individuals who have taken ivermectin before and TaNT at the health area or district level for those requiring testing. Such an approach would further reduce the time to elimination and costs. Generally, the number of people who (after participating in TaNT) take ivermectin is crucial for onchocerciasis elimination.

Alternative treatment strategies might be considered particularly in areas with higher precontrol onchocerciasis mf levels because those areas would require more effort on the part of programs to ensure good participation rates and minimal systematic nonparticipation. Mass treatment with a drug that is effective for onchocerciasis and safe to use in people with high loiasis MFD would be programmatically preferred. For example, drugs that work by killing the *Wolbachia* symbiont of *O volvulus* worms (not present in *L loa* worms) or other macrofilaricidal only or both micro- and macrofilaricidal drugs could be considered. An example is doxycycline, which is known to have macrofilaricidal activity against *O volvulus* but does not affect *L loa* [23, 24]. Unfortunately, doxycycline is impractical to use on a large scale, because of the long regimen (4-6 weeks) and contraindications in pregnant women and children younger than 12 years of age. Alternatively, a macrofilaricide could be provided only to individuals excluded from ivermectin because of TaNT and after testing for *O volvulus*. Such an approach would lower the transmission intensity and would likely decrease the required time to elimination of onchocerciasis. This would only be practical if the percentage of exclusion were low. To further reduce the time to elimination, complementary vector control might be considered as an additional measure [25].

As in previous studies, we defined elimination as reaching a modeled mf prevalence below 1.4%. Whether the chosen threshold would lead to elimination of transmission strongly depends on precontrol mf levels and local transmission conditions [26]. At lower transmission intensities, a low threshold may not be necessary to achieve elimination. In fact, elimination of transmission, defined as 99% probability of elimination 50 years posttreatment, could be achieved after 5 to 6 years of treatment in hypoendemic settings, meaning that elimination could already be achieved at a higher mf prevalence threshold than the suggested 1.4% (Figure S4). This threshold also depends on the assumed level exposure heterogeneity. When a lower level of exposure heterogeneity is assumed, elimination could be achieved sooner. The 1.4% threshold used in this study can be regarded as conservative, and it is very likely that elimination of transmission would be achieved when this threshold is reached.

In reality, the infection dynamics will be influenced by movement of infected humans or flies, as well as changes in demographic, geographic, and environmental conditions. To account for movement of infected humans or flies, our model includes an external force of infection representing incoming infections from neighboring areas. The level of control in neighboring areas is an important determinant for the success and duration of elimination programs in a certain village. If treatment (eg, ivermectin MDA) in neighboring areas occurred earlier, time to elimination would drop by 4 to 6 years (Supplementary Figure S5). Moreover, if we assume no incoming infections from neighboring villages, elimination could be achieved without intervention in some situations.

## CONCLUSIONS

Our model predicts that onchocerciasis can be eliminated using TaNT in hypoendemic areas co-endemic for *L loa*. Assuming good participation, the required duration of TaNT to reach a threshold of 1.4% mf prevalence would be only slightly longer than in areas with conventional ivermectin MDA. It will be most challenging to achieve elimination in areas that are in the upper end of the hypoendemic profile. In such areas, elimination could take more than 15 years depending on participation and TaNT exclusion rates.

## Supplementary Data

Supplementary materials are available at *Clinical Infectious Diseases* online. Consisting of data provided by the authors to benefit the reader, the posted materials are not copyedited and are the sole responsibility of the authors, so questions or comments should be addressed to the corresponding author.

ciaa1829_suppl_Supplementary_Material

## Notes

***Author contributions.*** D. B., J. K., S. P., H. N.-D., Y. N.-E., C. C., C. M., A. K., D. F., T. N., S. d. V., M. B., and W. S. contributed to the planning of the study, interpretation of the results, and reporting of the work. D. B. and W. S. conducted the analysis. All authors have read and approved the manuscript.

***Financial support.*** This work was supported by the Bill and Melinda Gates Foundation (grant numbers TaNT project OPP1033740, NTD Modelling Consortium OPP1184344) and in part by the Division of Intramural Research (National Institute of Allergy and Infectious Diseases, US National Institutes of Health). The funding source had no role in the study design, analysis, interpretation, or writing of the report. The corresponding author had full access to all the data in the study and had final responsibility for the decision to submit for publication.

***Potential conflicts of interest.*** D. F. has patents for High Numerical Aperture Telemicroscopy Apparatus (US Patents 8 743 194 and 8 786 695), licensed to Thermo Fisher Scientific. All other authors declare no competing. All other authors report no potential conflicts.
