# Supplementary material for: Feasibility of Onchocerciasis Elimination Using a “Test-and-not-treat” Strategy in Loa loa Co-endemic Areas
Source: Clin Infect Dis. 2020 Dec 8;72(12):e1047–55. doi: 10.1093/cid/ciaa1829 (PMC8204788; doi:10.1093/cid/ciaa1829)
Supplement: ciaa1829_suppl_Supplementary_Material [file ciaa1829_suppl_supplementary_material.docx]

**Supplementary Data**

**Contents**

[Supplementary Table 1. ONCHOSIM quantification used to simulate onchocerciasis transmission. 2](#_Toc57022463)

[Supplementary Table 2. Modeled scenarios 5](#_Toc57022464)

[Supplementary Table 3. The Policy-Relevant Items for Reporting Models in Epidemiology of Neglected Tropical Diseases (PRIME-NTD) ^a^ 6](#_Toc57022465)

[Supplementary Figure 1. Parameters combinations used to simulate the baseline pre-control onchocerciasis mf prevalence. 7](#_Toc57022466)

[Supplementary Figure 2. Distribution of intensity of *O. volvulus* infection. 8](#_Toc57022467)

[Supplementary Figure 3. Modeled reduction of external force of *O. volvulus* infection. 9](#_Toc57022468)

[Supplementary Figure 4. Probability of onchocerciasis elimination at 50 years post-treatment. 10](#_Toc57022469)

[Supplementary Figure 5. Average time needed to reach an onchocerciasis mf prevalence below 1.4% assuming earlier start of treatment in neighboring villages. 11](#_Toc57022470)

# Supplementary Table 1. ONCHOSIM quantification used to simulate onchocerciasis transmission.

| **Parameter** | **Value** | **Source** |
| --- | --- | --- |
| **Transmission of infection** | | |
| *General transmission parameters* |  |  |
| Relative biting rate (*rbr*) | Multiplied with the reference *mbr* values, to modify the monthly and annual biting rate: varied between simulations. |  |
| Seasonal variation in contribution to reservoir (*mbr*) | Reference *mbr* values (Jan-Dec):  5340, 4700, 3000, 3840, 3880, 3380,  5260, 6820, 6020, 6580, 7500, 5380 | Alley and colleagues^1^ & Entomological data collated by OCP |
| Transmission probability (*v*), i.e. the probability that an infective particle in the reservoir successfully develops into a parasite life stage that is capable of infecting a human host | *v* = 0.07345; see reference for the derivation of this value, given parameters for fly biology and development of infective L3 larvae within the fly. | Coffeng and colleagues^2^ |
| Success ratio (*sr*) | *sr* = 0.0031 | Duke^3^ & Plaisier^4^ |
| Zoophily (*z, 1 -h*) | *z* = 0.04 ; *h* = 0.96 | Habbema and colleagues^5^ & expert opinion (OCP entomologists) |
| *Individual relative exposure and contribution to flies* | |  |
| Relative exposure and contribution by age and sex | Zero at birth, linearly increasing between ages 0–20 from 0 to 1.0 for men and from 0 to 0.7 for women, and then constant from the age of 20 years onwards | Plaisier^4^ |
| Variation due to personal factors (fixed through life) given age and sex (*α_Exi_*) | Gamma distribution with mean 1.0. Shape and rate equal varies between simulations |  |
| **Life history and productivity of the parasite in the human host** | | |
| Average worm lifespan (*Tl*) | 10 years | Plaisier and colleagues^6^ |
| Variation in worm lifespan | Weibull distribution with shape 3.8. | Assumption; Plaisier and colleagues^6^ |
| Prepatent period (*pp*) | 1 year | Plaisier and colleagues^6^ which refers to Duke^7^ & Prost^8^ |
| Age-dependent microfilaria production capacity, *R(a)* | *R(a)* = 1 for 0 ≤ *a* < 5 | Plaisier and colleagues^6^ which refers to Albiez^9^ & Karam and colleagues^10^ |
|  | *R(a)* = 1-((a-5)/15) for 5 ≤ *a* < 20 |  |
|  | *R(a)* = 0 for *a* > 20 |  |
| Longevity of microfilariae within host (*Tm*) | 9 months | Plaisier^4^ |
| Mating cycle (*rc*) | 3 months | Plaisier^4^ & Plaisier and colleagues^6^ which refers to Schulz-Key^11^ & Schulz-Key and Karam^12^ |
| Male potential (*pot*) | 100 female worms. | Plaisier ^4^ |
| *Density-dependent female worm reproductive capacity* | |  |
| Average contribution of an inseminated worm at peak fecundity to the skin mf-density | 7.6 mf/worm | Plaisier^4^ |
| Exponential saturation of individual female worm productivity per worm present in host (*λ_z_*) | *λ_z_* = 0 i.e. no exponential saturation. | Assumption |
| **Morbidity** | | |
| Disease threshold (*Elc*) for blindness | Weibull distribution with mean 10.000 and shape 2.0 | Coffeng and colleagues^13^ |
| Reduction in remaining life expectancy due to blindness (*rl*) | 50% | Coffeng and colleagues^13^ which refers to partly published data from OCP; Dadzie and colleagues^14^;  Plaisier and colleagues^15^ which refers to Kirkwood and colleagues^16^ & Prost and Vaugelade^17^ |
| **Infection dynamics in the vector** | | |
| L1-uptake in the vector | Exponential saturating function with parameters *a* = 1.2, *b* = 0.0213, and *c* = 0.0861. | Plaisier and colleagues^18^ which refers to Philippon^19^ & WHO^20^ |
| **Mass treatment coverage** | | |
| Timing and coverage (*C_w_*_­_) | Varied between scenarios |  |
| Relative compliance (c_r_(k, s)) by age and sex   \| Age-group (k) \| *0-4* \| *5-9* \| *10-14* \| *15-19* \| *20-29* \| *30-49* \| *50+* \| \| --- \| --- \| --- \| --- \| --- \| --- \| --- \| --- \| \| cr(k,males) \| *0.00* \| *0.75* \| *0.80* \| *0.80* \| *0.70* \| *0.75* \| *0.80* \| \| cr(k,females) \| *0.00* \| *0.75* \| *0.70* \| *0.74* \| *0.65* \| *0.70* \| *0.75* \| | | Based on unpublished OCP data |
| **Drug treatment** | | |
| Proportion of microfilariae cleared from host | 100% | Plaisier and colleagues^21^ |
| Duration of temporary reduction in female reproductive capacity (*Tr_0_*), average | 11 months | Plaisier and colleagues^21^ |
| Permanent reduction in female worm reproductive capacity (*d*_0_), average | 34.9% | Plaisier and colleagues^21^ |
| Proportion of adult worms killed (*m*_0_) | 0% | Plaisier and colleagues^21^ |
| Relative effectiveness of treatment in a person (*v*) | Weibull distribution with mean 1 and shape 2 | Plaisier and colleagues^21^ |
| **Surveys** |  |  |
| Timing | Surveys are done at yearly intervals from 1988-2076. They are always done in month 6, i.e. exactly 12 or 6 months after annual or biannual treatment respectively. The simulation allows for a 200-year warming-up period before the first survey in 1998. |  |
| Dispersal factor for worm contribution to measured density of infective material (*d*) | Exponential distribution with mean 1 | Plaisier and colleagues^6^ |
| Variability in measured host load of infective material (here: mf per skin snip) | Poisson distribution with mean $ss\left( t \right)$ | Plaisier and colleagues^15^ |

**References**

1. Alley ES, Plaisier AP, Boatin BA, et al. The impact of five years of annual ivermectin treatment on skin microfilarial loads in the onchocerciasis focus of Asubende, Ghana. *Trans R Soc Trop Med Hyg* 1994; **88**(5): 581-4.

2. Coffeng LE, Stolk WA, Hoerauf A, et al. Elimination of African onchocerciasis: modeling the impact of increasing the frequency of ivermectin mass treatment. *PLoS One* 2014; **9**(12): e115886.

3. Duke BO. The population dynamics of Onchocerca volvulus in the human host. *Trop Med Parasitol* 1993; **44**(2): 61-8.

4. Plaisier AP. Modelling onchocerciasis transmission and control. Rotterdam, The Netherlands: Erasmus University; 1996.

5. Habbema JDF, van Oortmarssen GJ, Plaisier AP. The ONCHOSIM model and its use in decision support for river blindness control. In: Isham V, Medley G, eds. Models for infectious human diseases Their structure and relation to data. Cambridge, UK: Cambridge University Press; 1996: 360-80.

6. Plaisier AP, van Oortmarssen GJ, Remme J, Habbema JD. The reproductive lifespan of Onchocerca volvulus in West African savanna. *Acta Trop* 1991; **48**(4): 271-84.

7. Duke BO. Observations on Onchocerca volvulus in experimentally infected chimpanzees. *Tropenmed Parasitol* 1980; **31**(1): 41-54.

8. Prost A. Latence parasitaire dans I'onchocereose. *Bull World Health Organ* 1980; **58**: 923-5.

9. Albiez EJ. Calcification in adult Onchocerca volvulus. *Trop Med Parasitol* 1985; **36**(3): 180-1.

10. Karam M, Schulz-Key H, Remme J. Population dynamics of Onchocerca volvulus after 7 to 8 years of vector control in West Africa. *Acta Trop* 1987; **44**(4): 445-57.

11. Schulz-Key H. Observations on the reproductive biology of Onchocerca volvulus. *Acta Leiden* 1990; **59**(1-2): 27-44.

12. Schulz-Key H, Karam M. Periodic reproduction of Onchocerca volvulus. *Parasitol Today* 1986; **2**(10): 284-6.

13. Coffeng LE, Stolk WA, Zoure HG, et al. African Programme For Onchocerciasis Control 1995-2015: model-estimated health impact and cost. *PLoS Negl Trop Dis* 2013; **7**(1): e2032.

14. Dadzie KY, Remme J, Rolland A, Thylefors B. The effect of 7-8 years of vector control on the evolution of ocular onchocerciasis in West African savanna. *Trop Med Parasitol* 1986; **37**(3): 263-70.

15. Plaisier AP, van Oortmarssen GJ, Habbema JD, Remme J, Alley ES. ONCHOSIM: a model and computer simulation program for the transmission and control of onchocerciasis. *Comput Methods Programs Biomed* 1990; **31**(1): 43-56.

16. Kirkwood B, Smith P, Marshall T, Prost A. Relationships between mortality, visual acuity and microfilarial load in the area of the Onchocerciasis Control Programme. *Trans R Soc Trop Med Hyg* 1983; **77**(6): 862-8.

17. Prost A, Vaugelade J. La surmortalité des aveugles en zone de savane ouest-africaine. *Bull World Health Organ* 1981; **59**: 773-6.

18. Plaisier AP, van Oortmarssen GJ, Remme J, Alley ES, Habbema JD. The risk and dynamics of onchocerciasis recrudescence after cessation of vector control. *Bull World Health Organ* 1991; **69**(2): 169-78.

19. Philippon B. Etude de la transmission d' Onchocerca volvulus (Leuckart, 1983) Nematoda, Onchocercidae) par Simulium damnosum (Theobald, 1903) (Diptera, Simuliidae) en Afrique tropicale. *Travaux et Documents ORSTOM* 1977; **63**.

20. World Health Organization. Onchocerciasis Control Programme in West Africa: report of the annual OCP research meeting. 1989.

21. Plaisier AP, Alley ES, Boatin BA, et al. Irreversible effects of ivermectin on adult parasites in onchocerciasis patients in the Onchocerciasis Control Programme in West Africa. *J Infect Dis* 1995; **172**(1): 204-10.

# Supplementary Table 2. Modeled scenarios

| **Scenarios** | **Participation rate** | **Systematic non-participation** | **Exclusion rate due to high L. Loa MFD ^a^** |
| --- | --- | --- | --- |
| A | 80% | 0% | 1%, 2.5%, 5%, 7.5% and 10% |
| B | 65% | 0% | 1%, 2.5%, 5%, 7.5% and 10% |
| C | 65% | 5% | 1%, 2.5%, 5%, 7.5% and 10% |
| D | 65% | 10% | 1%, 2.5%, 5%, 7.5% and 10% |
| E | 50% | 0% | 1%, 2.5%, 5%, 7.5% and 10% |
| F | 50% | 5% | 1%, 2.5%, 5%, 7.5% and 10% |
| G | 50% | 10% | 1%, 2.5%, 5%, 7.5% and 10% |
| ^a^ Rate of exclusion from ivermectin due to high *L. Loa* microfilarial densities (MFD) was calculated as: (1-Exclusion rate) * participation rate. The percentage excluded from treatment due to high *L. Loa* MFD was modelled similarly to systematic non-participation. | | | |

# Supplementary Table 3. The Policy-Relevant Items for Reporting Models in Epidemiology of Neglected Tropical Diseases (PRIME-NTD) ^a^

| **Principle ^b^** | **What has been done to satisfy the principle?** | **Where in the manuscript is this described?** |
| --- | --- | --- |
| 1. Stakeholder engagement | Key stakeholders are policy makers of oncho endemic – loa co-endemic countries. Representatives of that group are included in the TaNT project group and author list of this paper | - |
| 1. Complete model documentation | The paper contains a brief description of the model with references to the model and original model description papers. We provide information on the quantification of transmission, life history and productivity of the parasite, morbidity, vector, drug efficacy, treatment coverage and survey. | Methods section and table S1 |
| 1. Complete description of data used | Data and parameters used have been described in the manuscript and supplementary material. Most parameters in the model rely on quantification from previous published studies. | Methods section, table S1, figure S1 and S2 |
| 1. Communicating uncertainty | In addition to parameter uncertainty (see figure 1 and 3), we also conducted several sensitivity analyses: 1) allowing exclusion due to high *L. loa* loads to decrease over time; 2) changing start year of drop in external force of infection; 3) assessing the probability of elimination of transmission 50 years post-treatment by exposure heterogeneity. | Results section, figure 1, 3, 4, S4 and S5 |
| 1. Testable model outcomes | Model outcomes include mf trends, time to elimination, and the probability of elimination. The first two outcomes can be compared with other models. | Results section, table 1, figure, 1,2, and 3 |
| ^a^ Communication of adherence to the five principles of the NTD Modelling Consortium for policy-relevant work, described in: Behrend et al. 2020. Modelling for policy: The five principles of the Neglected Tropical Diseases Modelling Consortium. *PLoS Negl Trop Dis* 2020; **14**(4): e0008033.  ^b^ Full formulation of the principles:   1. Don't do it alone. Engage stakeholders throughout, from the formulation of questions to the discussions on the implications of the findings. 2. Reproducibility is key! Prepare and make available (preferably open-source) a complete technical documentation of all model code, mathematical formulas, assumptions and their justification, allowing others to reproduce the model. 3. Model calibration, goodness-of-fit and validation are fundamental processes of scientific modelling. All data used should be described in sufficient detail to allow the reader to assess the type and quality of these analyses. When using data by reference, use Principle 2. 4. Communicating uncertainty is a hallmark of good modelling practice. Perform a sensitivity analysis of all key parameters, and for each paper reporting model predictions include an uncertainty assessment of those model outputs within the paper. 5. Model outcomes should be articulated in the form of testable hypotheses. This allows comparison with other models and future events as part of the ongoing cycle of model improvement. | | |


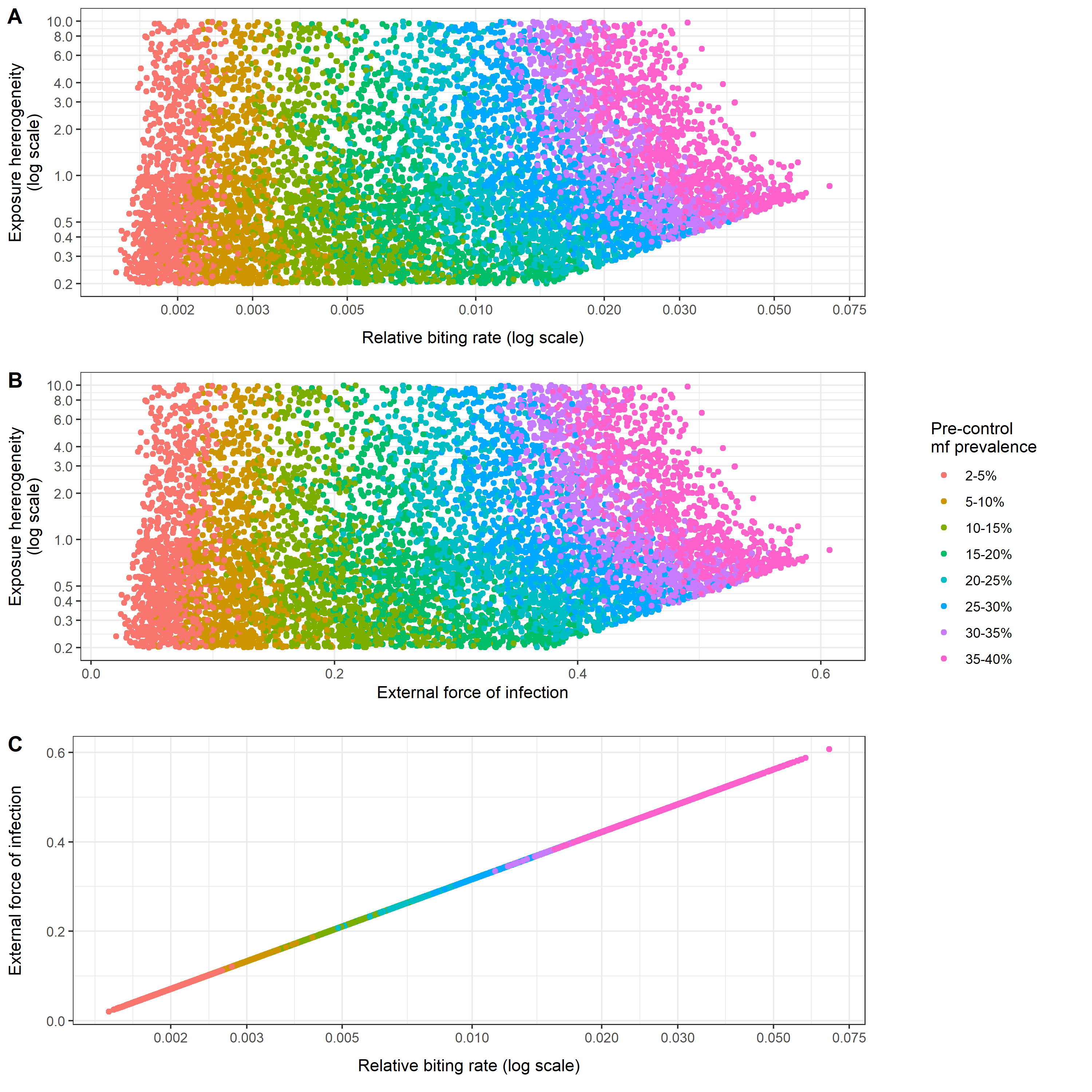


**Supplementary Figure 1. Parameters combinations used to simulate the baseline pre-control onchocerciasis mf prevalence.** Results of sampling annual biting rate, exposure heterogeneity and external force of infection from a predefined parameter space. Each parameter combination reflects a pre-control mf prevalence level. The colors represent the pre-control onchocerciasis mf prevalence bin to which a parameter combination belongs. Each bin contains 1,000 parameter combinations (8,000 in total).


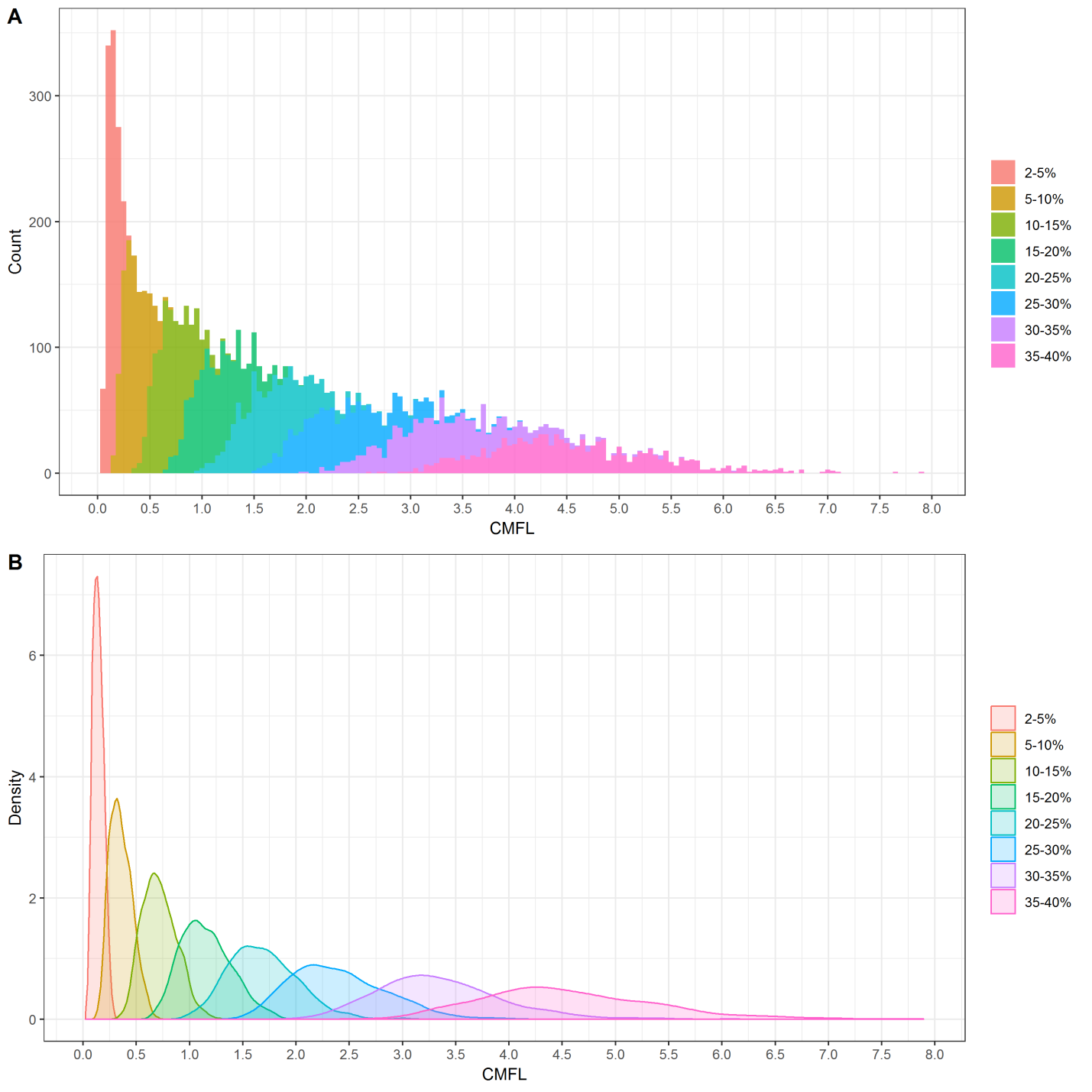


**Supplementary Figure 2. Distribution of intensity of *O. volvulus* infection.** A) Frequency distribution of the community microfilarial load (CMFL); B) Density of CMFL. The colors represent the pre-control onchocerciasis mf prevalence bin.


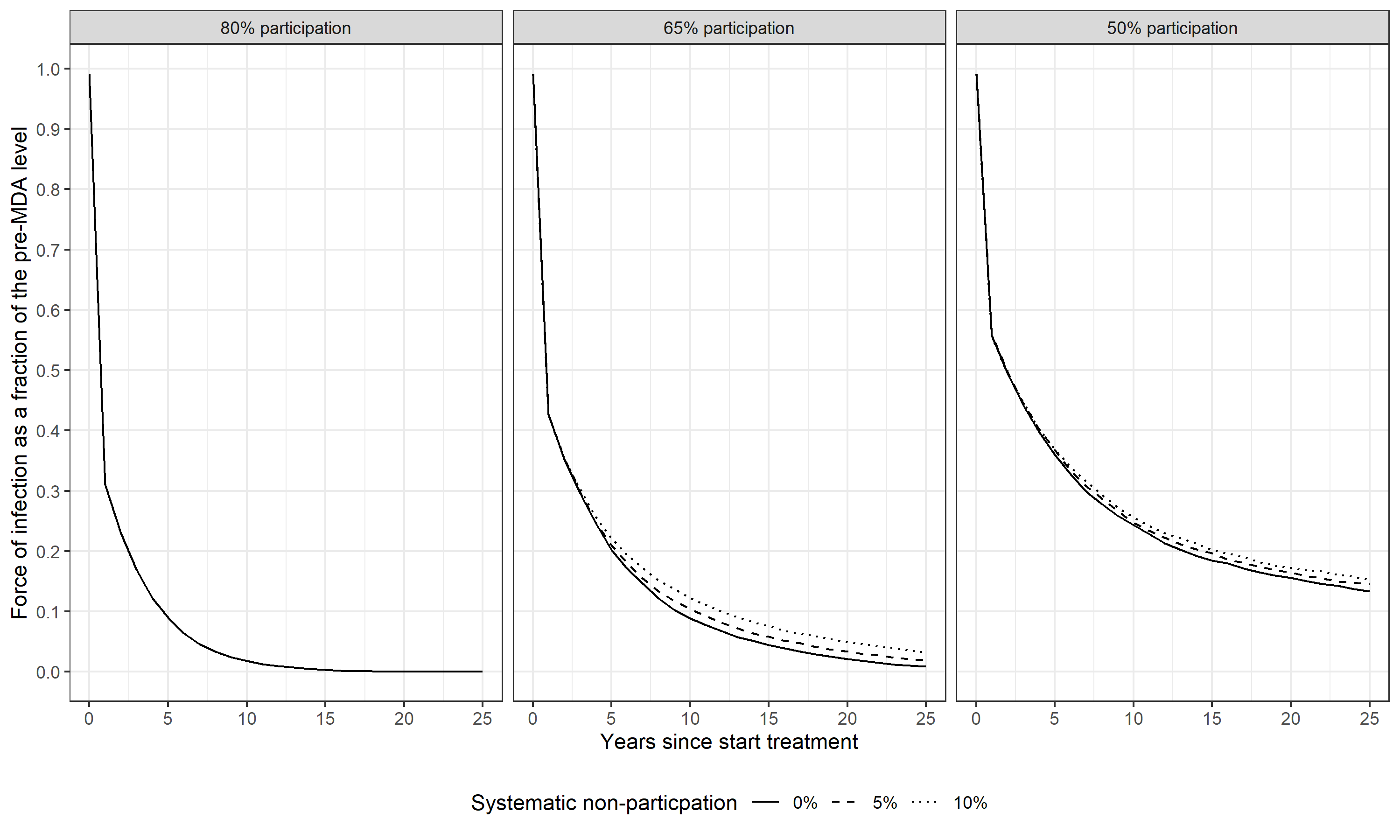


**Supplementary Figure 3. Modeled reduction of external force of *O. volvulus* infection.** The rate of decline in the external force of infection was estimated by simulating a hyperendemic area with annual ivermectin MDA. The resulted drop in the force of infection is considered as a proxy of the rate of decline in the external force of infection.


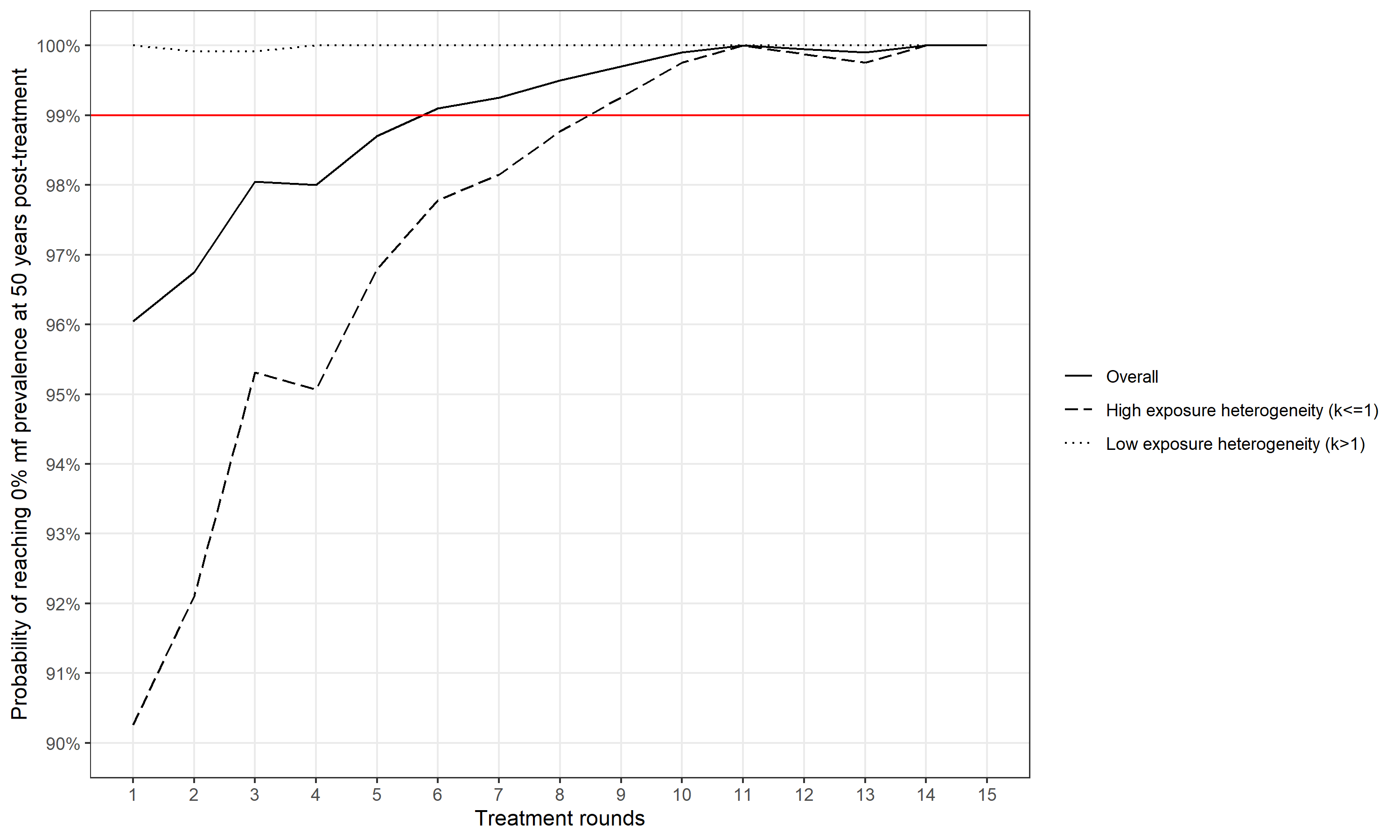


**Supplementary Figure 4. Probability of onchocerciasis elimination at 50 years post-treatment.** Elimination is defined as reaching 0% onchocerciasis mf prevalence at 50 years post-treatment. The pre-control onchocerciasis mf prevalence is 30-40%. The solid black line shows the overall results; the dashed and dotted black lines show the results by high and low exposure heterogeneity, respectively. The red line is the 99% probability of reaching elimination at 50 years post-treatment. The overall results show that achieving elimination with 99% probability could be achieved after 5 to 6 treatment rounds. This would correspond to a mf prevalence threshold of 10.9-13.3%. The required number of treatment rounds would be higher if exposure heterogeneity is high (dashed line).


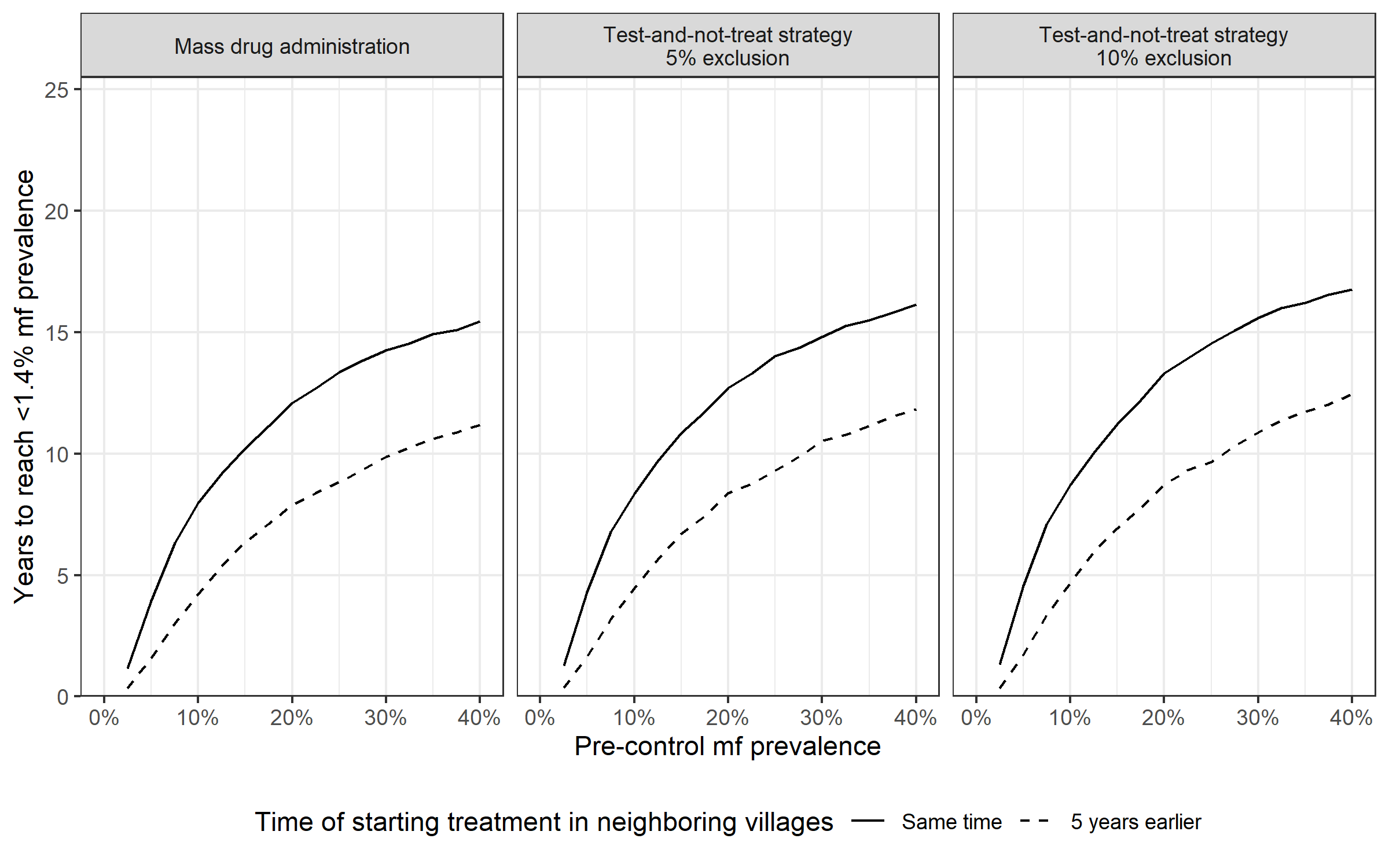


**Supplementary Figure 5. Average time needed to reach an onchocerciasis mf prevalence below 1.4% assuming earlier start of treatment in neighboring villages.** We assumed 65% participation with 5% systematic non-participation. The panels indicate the different strategies: ivermectin MDA (i.e. without pre-testing) and test-and-not-treat assuming 5 and 10% exclusion due to high *L. loa* microfilarial densities. The solid line represents a situation where treatment in neighboring areas starts at the same time as in the modeled area. The dashed line represent a situation where treatment in neighboring areas have started 5 years earlier, i.e. the external force of infection started to decrease 5 years prior the start of treatment in the modeled area.
